# Physiological Analysis and Genetic Mapping of Short Hypocotyl Trait in *Brassica napus* L.

**DOI:** 10.3390/ijms242015409

**Published:** 2023-10-21

**Authors:** Miaomiao Liu, Fangdi Hu, Lijun Liu, Xiaoming Lu, Rong Li, Jingyu Wang, Junyan Wu, Li Ma, Yuanyuan Pu, Yan Fang, Gang Yang, Wangtian Wang, Wancang Sun

**Affiliations:** State Key Laboratory of Aridland Crop Science, College of Agronomy, Gansu Agricultural University, Lanzhou 730070, China; 15193822285@163.com (M.L.);

**Keywords:** *Brassica napus* L., physiological and biochemical parameters, hypocotyl length, BSA-seq

## Abstract

Hypocotyl length is a botanical trait that affects the cold tolerance of *Brassica napus* L. (*B. napus*). In this study, we constructed an F_2_ segregating population using the cold-resistant short hypocotyl variety ‘16VHNTS158’ and the cold-sensitive long hypocotyl variety ‘Tianyou 2288’ as the parents, and BSA-seq was employed to identify candidate genes for hypocotyl length in *B. napus*. The results of parental differences showed that the average hypocotyl lengths of ‘16VHNTS158’ and ‘Tianyou 2288’ were 0.41 cm and 0.77 cm at the 5~6 leaf stage, respectively, after different low-temperature treatments, and ‘16VHNTS158’ exhibited lower relative ion leakage rates compared to ‘Tianyou 2288’. The contents of indole acetic acid (IAA), gibberellin (GA), and brassinosteroid (BR) in hypocotyls of ‘16VHNTS158’ and ‘Tianyou 2288’ increased with decreasing temperatures, but the IAA and GA contents were significantly higher than those of ‘Tianyou 2288’, and the BR content was lower than that of ‘Tianyou 2288’. The genetic analysis results indicate that the genetic model for hypocotyl length follows the 2MG-A model. By using SSR molecular markers, a QTL locus associated with hypocotyl length was identified on chromosome C04. The additive effect value of this locus was 0.025, and it accounted for 2.5% of the phenotypic variation. BSA-Seq further localized the major effect QTL locus on chromosome C04, associating it with 41 genomic regions. The total length of this region was 1.06 Mb. Within this region, a total of 20 non-synonymous mutation genes were identified between the parents, and 26 non-synonymous mutation genes were found within the pooled samples. In the reference genome of *B. napus*, this region was annotated with 24 candidate genes. These annotated genes are predominantly enriched in four pathways: DNA replication, nucleotide excision repair, plant hormone signal transduction, and mismatch repair. The findings of this study provide a theoretical basis for cloning genes related to hypocotyl length in winter rapeseed and their utilization in breeding.

## 1. Introduction

The hypocotyl is the beginning stem portion of the plant below the cotyledonary insertion, the embryonic organ connecting the cotyledon and root, and it is also the demarcation area between the stem and root. Hypocotyl elongation ensures the smooth transportation of water, inorganic salts, nutrients, and hormones [1], assisting in the emergence of seedlings from the soil and the transition from heterotrophic to autotrophic growth; however, elongation of the hypocotyl often leads to elongated and weak seedlings with poor stress resistance [2]. Therefore, appropriate hypocotyl length is essential for normal plant activities [3], playing a significant role in plant responses to adversity stress [4]. *Brassica napus* L. (*B. napus*) is a globally important oil-seed crop, and the regulatory mechanism of hypocotyl length and plant cold tolerance can be investigated to analyze the adaptation of *B. napus* under low-temperature adversity, especially in the northern regions where winter conditions are extremely cold and dry, and winter rapeseed faces harsh wintering conditions. Therefore, studying and deciphering the regulatory mechanisms of hypocotyl length in winter rapeseed have significant theoretical and practical implications.

It has been shown that hypocotyl length is regulated by the integration of environmental information and signaling pathways involved in the regulation of seedling development, including the photoreceptors phytochrome A (PHYA), phytochrome B (PHYB), circadian cryptochrome 1 (CRY1), and circadian cryptochrome 2 (CRY2) [5,6,7], as well as YUCCA (YUC) protein and DELLA protein, which collectively regulate the phytochrome-interacting factors (PIFs), affecting the expression of the downstream genes to control hypocotyl elongation [8,9,10]. Under light conditions, cytokinin interacts with the ethylene signaling pathway to synergistically regulate hypocotyl elongation [11]. Studies on the localization of hypocotyl-length genes have been reported, but so far, no exact genes have been cloned, and fewer molecular markers have been used for cold tolerance breeding practices. Previous studies have analyzed three long hypocotyl lines and three short hypocotyl lines in a doubled haploid (DH) population through whole-genome association analysis and transcriptome sequencing. It was discovered that *BnaC07g46770D* directly negatively regulates hypocotyl elongation [12]. The continuous development of high-throughput sequencing technologies has provided an effective means for rapid gene discovery and functional marker development. BSA-seq technology can rapidly mine target genes without constructing a genetic map, and this technology has been widely used in the study of many crops [13], successfully localizing genes such as those associated with traits, such as *B. napus* chloroplast development, peanut seed coat [14], cabbage white flowers [15], and rice nematodes [16]. In the northern regions, winter rapeseed undergoes the gradual cessation of aboveground and underground growth before winter [17]. A longer hypocotyl in general leads to the exposure of the apical meristem to the atmosphere, rendering the plants susceptible to freezing injury [18]. Therefore, breeding a winter rapeseed variety with a short hypocotyl is of particular importance. In this study, using BSA-seq technology combined with SSR molecular markers, we employed the short hypocotyl variety ‘16VHNTS158’, the long hypocotyl variety ‘Tianyou 2288’, and the hybrid offspring to screen key genes that can respond to low-temperature stress and regulate hypocotyl elongation. The purpose is to provide insights and references for the precise positioning of the short hypocotyl gene, as well as to lay a foundation for the further cloning of genes to control short hypocotyls, the elucidation of the regulatory mechanisms of related pathways, and the breeding of new varieties of cold-resistant winter rapeseed.

## 2. Results

### 2.1. Physiological Basis of Plant Responses to Low-Temperature Stress

The lethal temperature (LT50) is an important index reflecting the strength of plant cold tolerance, and when the treatment low temperature is lower than LT50, the plant will be irreversibly injured, and the relative conductivity will increase rapidly. The LT50 values of ‘16VHNTS158’ and ‘Tianyou 2288’ were −7.5 °C and −5.4 °C, respectively (Table 1).

**Figure 1:**
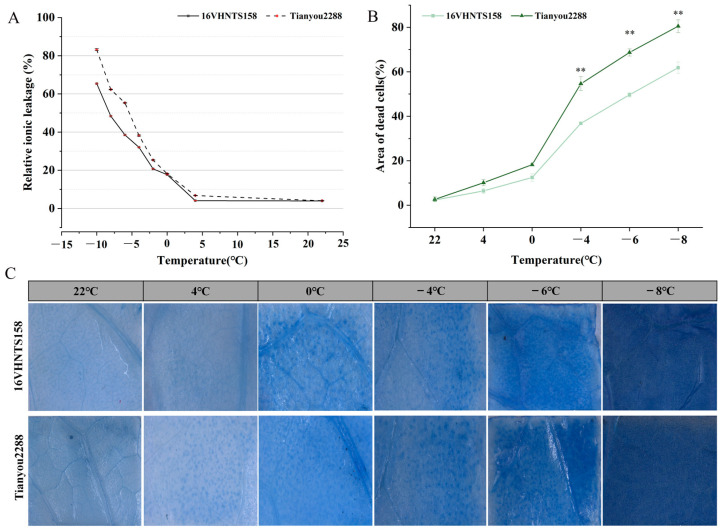
Changes in ion leakage rate and cell death area in plants under low-temperature stress. (**A**) Changes in ion leakage rate after low-temperature stress. (**B**) Statistics on the area of death of two parents. Asterisks indicate significant differences: ** *p* < 0.01. (**C**) Observation of Trypan blue staining of ‘16VHNTS158’ and ‘Tianyou 2288’. Bar = 1 mm.

To verify the difference in cold resistance between the cold-resistant variety and cold-sensitive variety, the ion leakage rates between the two parents after low-temperature treatments were measured and compared (Figure 1A). The results show that after different low-temperature treatments, the ion leakage rates of both ‘16VHNTS158’ and ‘Tianyou 2288’ showed a continuously increasing trend, and the ion leakage rate of the cold-resistant variety was lower than that of the cold-sensitive variety, ‘Tianyou 2288’, under all temperature treatments. Compared with the 22 °C treatment, the ion leakage rate of ‘16VHNTS158’ increased by 3.1%, 344.24%, 420%, and 702.89%, respectively, and that of ‘Tianyou 2288’ increased by 68.14%, 351.17%, 530%, and 848.5%, respectively, under treatments from 4 to −4 °C; the leakage rate of the two parents was elevated under treatments at −8 °C compared with −6 °C, at 221.1% and 154.8%, respectively, indicating that the cells in the plant leaves were essentially dead at −8 °C. These results suggest that inorganic ions absorbed and stored by plants from the outside, which increase the cytoplasmic osmotic pressure against different degrees of low-temperature chilling during stress, are also a protective mechanism for plants.

### 2.2. Observation of Trypan Blue Staining and Counting of Dead Cell Area

Trypan blue staining was used to observe the cells of ‘16VHNTS158’ and ‘Tianyou 2288’ leaves after different low-temperature treatments (Figure 1B). The results show that before being subjected to low-temperature treatment, the cells of ‘16VHTNS158’ and ‘Tianyou 2288’ leaves were not stained, which indicated that the cell viability of the leaves at 22 °C was in a good condition, and there was no cell death. After different degrees of low-temperature stress, with regard to stained cells in the leaves of the two varieties, the number of stained cells was higher in ‘Tianyou 2288’. The number of stained cells increased in the leaves of the two varieties after −8 °C treatment, and the cell membrane structure was seriously degraded. The number of dead cells in the leaves of ‘Tianyou 2288’ was much higher than that of ‘16VHNTS158’, and its dead area was 80.48%, whereas that of ‘16VHNTS158’ was 71.84% (Figure 1C).

The relationship between hypocotyl length and cold resistance was analyzed using LT50, dead cell area, and ion leakage rate as indicators for cold resistance assessment (Table S1). Hypocotyl length was found to have a highly significant positive correlation with LT50 and dead cell area, while it showed a highly significant negative correlation with ion leakage rate. Additionally, there was a highly significant negative correlation between dead cell area and ion leakage rate and a highly significant positive correlation between dead cell area and LT50.

### 2.3. Differences in Endogenous Hormone Content in Embryonic Axes under Low-Temperature Stress

**Figure 2:**
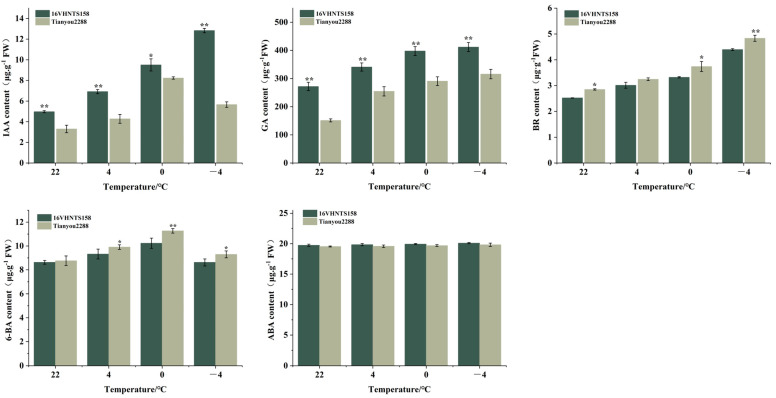
After low-temperature treatment, endogenous content of IAA, GA, BR, 6-BA, and ABA in the hypocotyl of ‘16VHNTS158’ and ‘Tianyou 2288’. Values represent means ± SD. Asterisks indicate significant differences: * *p* < 0.05, ** *p* < 0.01.

The results of endogenous hormone measurements (Figure 2) show that as the temperature decreased, the levels of indole acetic acid (IAA), gibberellins (GA), and brassinosteroids (BR) in the hypocotyls of ‘16VHNTS158’ and ‘Tianyou 2288’ gradually increased. Under all temperature treatments, the IAA and GA levels in ‘16VHNTS158’ were significantly or extremely significantly higher than those in ‘Tianyou 2288’. On the other hand, the BR and ABA levels in ‘16VHNTS158’ were lower than those in ‘Tianyou 2288’. These findings indicate that ‘16VHNTS158’ responds to low-temperature stress by synthesizing more IAA and GA in the hypocotyl. Moreover, the increase in IAA and GA levels in the hypocotyls of ‘16VHNTS158’ is relatively stable compared to ‘Tianyou 2288’, suggesting that *B. napus* with stronger cold resistance exhibits a more stable change in the levels of endogenous hormones in its hypocotyls. The four hormones may play an important role in the formation of hypocotyl morphology.

### 2.4. Measurement and Genetic Analysis of Hypocotyl Length in the Parents and F_2_ Populations

**Figure 3:**
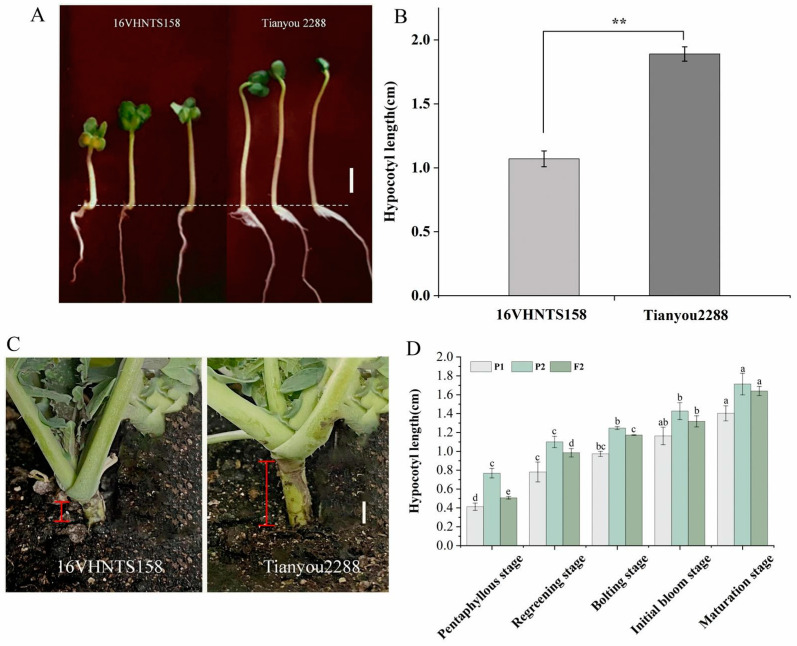
Morphological observation and length statistics of hypocotyls. (**A**) Observation of hypocotyl morphology in cotyledon stage. Bar = 3 cm. (**B**) Comparison of parental hypocotyl length. Asterisks indicate significant differences: ** *p* < 0.01. (**C**) Differences in hypocotyl length among parents at the 5-leaf stage. Bar =10 cm. White line represent 1cm, red line represent hypocotyl length. (**D**) Comparison of hypocotyl length at different developmental stages. P1 and P2 represent ‘16VHNTS158’ and ‘Tianyou 2288’, respectively. Letters represent significant difference analysis.

**Figure 4:**
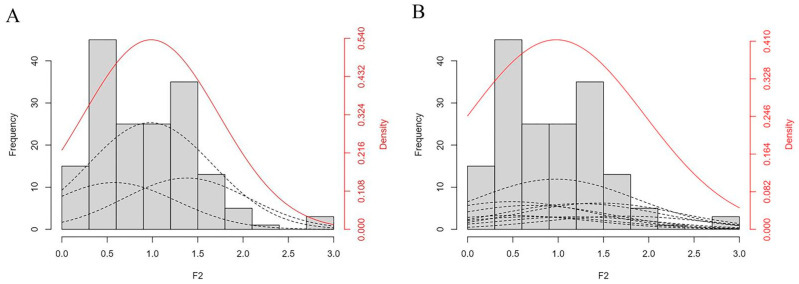
The frequency distribution of hypocotyl length in the F_2_-1 population under two different genetic models. (**A**) The frequency distribution of 1MG-A. (**B**) The frequency distribution of 2MG-A. Black dotted line represent genotype distribution in the (**A**,**B**).

The hypocotyl lengths of the F_2_-1 population (*n* = 185) and the two parents ‘16VHNTS158’ (P1) and ‘Tianyou 2288’ (P2) at different reproductive stages (Figure 3A,C), as well as the histograms of hypocotyl lengths, were plotted (Figure 3B,D). The average hypocotyl lengths of ‘16VHNTS158’ were 0.41 cm and 0.78 cm and those of ‘Tianyou 2288’ were 0.77 cm and 1.1 cm at the 5-leaf and greening stages, respectively. The hypocotyl elongation rate of ‘16VHNTS158’ was slower than that of ‘Tianyou 2288’. The hypocotyl length of the F_2_-1 population conformed to a normal distribution and showed continuous variation (Figure 4A,B), and the lengths ranged from 0 cm to 4.4 cm (Table 2), with a coefficient of variation (CV) of 46%. The significant variation in hypocotyl length observed may be attributed to the low-temperature environment.

The alternative models for hypocotyl traits were tested using five statistical fitness quantities: $U_{1}^{2}$, $U_{2}^{2}$, $U_{3}^{2}$ (Homogeneity test), _n_W^2^ (Smirnov test), and D_n_ (Kolmogorov test) (Table 3). The selection of the best genetic model was based on the principle of the minimum AIC value and minimum statistical significance levels and the number of statistical significance levels of the two models for hypocotyl length, where the 2MG-A model had a small AIC value relative to the 1MG-A model. Therefore, we selected 2MG-A as the best genetic model for hypocotyl length. The results of genetic parameters for the optimal genetic model for hypocotyl traits were calculated (Table 4), and the additive effect value for hypocotyl length was 0.22 cm (Figure S2), with a heritability of 59.54%, and the environmental variance accounted for 40.46%.

### 2.5. Mutant Locus Mapped on C04 Chromosome

**Figure 5:**
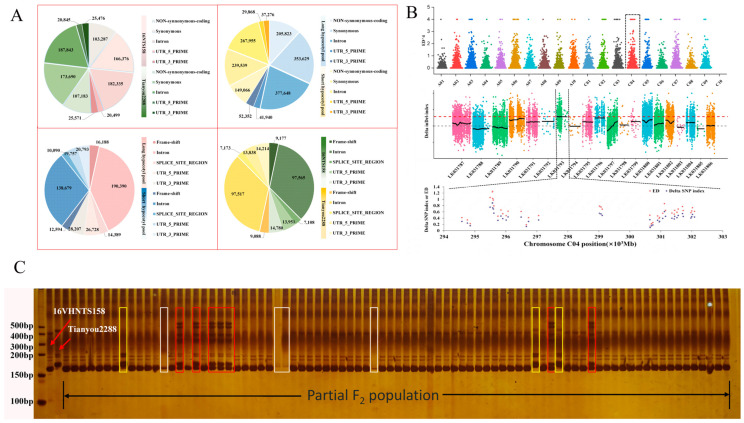
Mapping of the target gene using BSA-seq, and the screening of polymorphism using SSR molecular markers. (**A**) SNP and Indel annotation classification of two parents and two pools. (**B**) BSA-seq used to identify the genomic region of *B. napus*. The black dashed line indicates that the region on chromosome C04 showed the ΔSNP index with the highest value. The red dashed line represent the threshold line at 98% confidence level. The black solid line represent delta indel-index value. (**C**) The amplification results of primer C04-2 in 185 F_2_-1 individuals. Red boxes represent heterozygous bands. Yellow boxes represent the same bands as the parent ‘Tianyou 2288’. White boxes represent missing or blurred bands. Unchecked boxes represent bands that are identical to the parent ‘16VHNTS158’.

The whole-genome resequencing results of SNPs and Indels within the candidate region for each pooled sample are shown in Table 5 and Figure 5. There are 20 non-synonymous SNPs between the parental lines and 26 non-synonymous SNPs between the pooled samples. Additionally, there are six frameshift Indels between the parental lines and seven frameshift Indels between the pool samples (Figure 5A). These SNPs and Indels are highly likely to be directly related to the trait.

After filtering the raw data, we obtained clean reads totaling 165.66 Gb, with a Q30 (%) value of ≥92.21% and GC content of ≥36.51% (Table 5). The sequencing results for the long hypocotyl pool and short hypocotyl pool were 203,088,138 and 199,081,960, respectively, accounting for 99.22% and 99.47% of the total sequencing reads. The sequencing coverage of the reference genome assembly reached 33× and 32×, respectively. After filtering out low-quality SNPs and Indels with missing or heterozygous loci in the parental lines, 832,833 high-quality SNPs/Indels were obtained in the parental lines and 228,940 in the two pools. To eliminate false-positive loci, the ΔSNP index values on the same chromosome were fitted using the SNPNUM method using the location of the markers on the genome, and, then, the regions above the thresholds were selected as the regions associated with the traits. By using the 99th percentile of the fitted ΔSNP index values, 41 regions were identified (Table S2), with a length of 1.06 Mb. The region on chromosome C04 showed the highest value (Figure 5B), which may represent the major-effect QTL controlling this trait. Meanwhile, we designed primers to validate the sequencing results, including the primer C04-2 in 185 F_2_-1 individuals shown in Figure 5C. The region had 24 genes annotated in the *B. napus* reference genome.

Using the genotype detection results of the F_2_-1 population and the QTL IciMapping 4.0 software, a genetic linkage map of the C04 linkage group was constructed (Figures S1 and S2). The total genetic distance was 489.71 cM, with an average distance of 32.65 cM. A QTL associated with hypocotyl length was identified on chromosome C04, between 42.74 and 71.79 cM (Figure S1 and Table S3). Through BSA-seq analysis, the target gene was further narrowed down to a region of 1.06 Mb.

### 2.6. Selection of Candidate Regions and Functional Annotation

**Figure 6:**
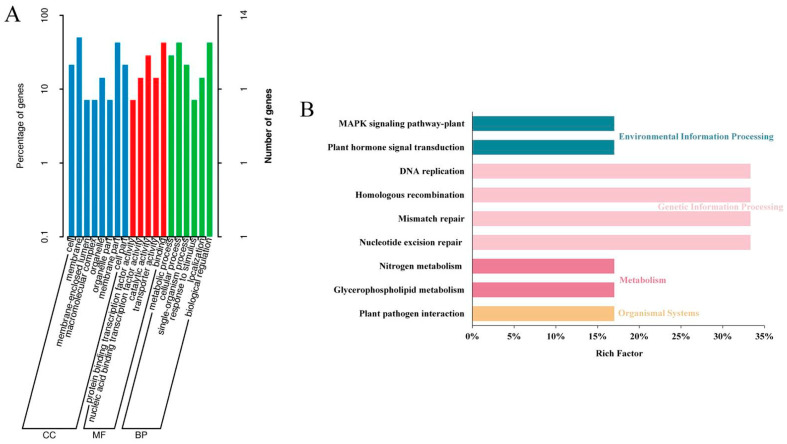
Functional analysis of the DEGs. (**A**) The GO terms in molecular function, cellular component, and biological process categories. (**B**) KEGG pathway enrichment of the DEGs.

To understand these differential candidate gene functions, GO enrichment analysis was performed, as shown in Figure 6A. Genes within the associated regions were classified into three ontological categories: molecular function, cellular component, and biological process. Specifically, 24 genes were enriched under the cellular component category (Table S4). In the biological process category, 22 genes were enriched under biosynthesis pathways, signal transduction pathways, metabolic pathways, specific responses to cytokinins, hormone stimulus, hormone-mediated signaling pathways, hormone metabolism pathways, cytokinin-mediated signaling pathways, responses to auxins, and negative regulation of biosynthetic processes. Fifteen genes associated with enzyme activities were enriched under the molecular function category and included protein-binding transcription factor activity, nucleotide-binding transcription factor activity, transport activity, catalytic activity, and binding activity. The possible roles of the genes in the metabolic pathways were determined via KEGG pathway analysis (Figure 6B). Enrichment analysis of differentially expressed genes in nine KEGG pathways revealed that DNA replication, nucleotide excision repair, homologous recombination, and mismatch repair were the four most enriched pathways, suggesting their significant roles in regulating underground plant structures in *B. napus*.

## 3. Discussion

The length of the hypocotyl is one of the structural phenotypes of *B. napus* plants. Short hypocotyl materials can exhibit cold resistance as a winter-avoidance mechanism, protecting the root growth cone from damage caused by low temperatures. However, the trait itself is not inherently cold-resistant. During seed germination to maturity, the hypocotyl elongates with developmental stages. The hypocotyl of cold-resistant materials elongates slower compared to those that are cold-sensitive. After cold stress, the hypocotyl continues to elongate but at a slower rate (Figure 3). This phenomenon was also mentioned by Zhang et al. [19]. However, under cold stress conditions (domestication and subzero temperatures), physiological changes in short hypocotyls show significant differences compared to long hypocotyls. Short hypocotyl plants exhibit lower LT50 and lower ion leakage rates, with smaller leaf cell death areas (Figure 1). As has been reported for Arabidopsis [20] and rice [21], with a mean LT50 of −21.8 °C when cold acclimated, plants display even lower LT50 and ion leakage rates, which is consistent with the findings of this study. These results seem to depend on the inherent cold resistance of the plants, indicating that the hypocotyl length and physiological response of winter rapeseed are influenced by specific growth conditions.

A change in the endogenous phytohormone is one of the most important resources for cellular protection against freezing. Its importance in freezing tolerance was demonstrated through the identification of four hormones significantly correlated with acclimatized freezing tolerance in *B. napus*, IAA, GA, ABA, and BR. Additionally, in the hypocotyl of *B. napus*, under low-temperature conditions, the accumulation of cold-responsive IAA, GA, ABA, and BR was observed (Figure 2). The pattern of change in these phytohormones was previously reported by Liu et al. [22] and Sadura et al. [23]. By contrast, we found that ABA levels appear to be independent of low temperature and variety, indicating that low temperature may reduce the sensitivity of the hypocotyl to abscisic acid (ABA). This phenomenon has been studied and reported in leguminous plants, where low temperature reduces the sensitivity of stomata to both endogenous and exogenous ABA, and the reason for this phenomenon may be attributed to the accumulation of ethylene induced by low temperature, which could mediate the insensitivity of stomata to ABA [24]. In this study, we screened for the *BnaC04g15990D* gene (Table S4), also known as lonely guy (log), which was previously identified as a cytokinin-activating enzyme that works in the direct activation pathway in rice (Oryza sativa) shoot meristems [25]. The responsible gene, *LOG*, encodes a cytokinin riboside 5′-monophosphate phosphoribohydrolase that releases cytokinin nucleobase and ribose 5′-monophosphate. LOG hydrolyzes only cytokinin riboside 5′-monophosphate but not AMP, suggesting that LOG is specifically involved in cytokinin activation [26]. However, it is not clear whether the direct activation pathway is biologically important in the hypocotyl of *B. napus* and whether there is functional differentiation between these two activating pathways.

Another BR hormone-related *BnaC04g16010D* gene identified in this study, a GSK3-like kinase (glycogen synthase kinase 3), also known as a SHAGGY-like kinase, is actively implicated in hormonal signal networks during development, as well as in biotic and abiotic stress responses [27]. Studies have found that *BnDF4* encodes brassinosteroid (BR)-insensitive 2, a glycogen synthase kinase 3 primarily expressed in the lower internodes to modulate rapeseed plant height by blocking basal internode cell elongation [28]. Therefore, we speculate that the *BnaC04g16010D* gene may also be involved in the biosynthesis and signal transduction of brassinosteroid (BR) plant hormones and have an inhibitory effect on hypocotyl cell elongation, leading to the manifestation of the short hypocotyl phenotype. These hypotheses need further research and validation.

Improving cold tolerance in winter rapeseed (*Brassica napus* L.) is an important breeding objective. Research on the response of rapeseed to low-temperature stress mainly includes pigment regulation [29], changes in enzyme system activity [26,30], protein expression [31], and gene function analysis [32]. However, there is limited research on the regulatory mechanism of the hypocotyl in the cold resistance of rapeseed. Furthermore, few studies have employed genetic methods to identify the genetic mechanisms underlying cold tolerance in the hypocotyl of *B. napus* [33]. A previously uncharacterized QTL, which is related to hypocotyl length under low-temperature stress, was identified using BSA-seq and SSR molecular markers. QTL mapping is a commonly used technique for dissecting complex quantitative trait loci in *B. napus*. For example, QTL mapping allows us to understand genetic regulation, including plant structure [34], yield [35], vegetative and oilseed types [36], shoot branching [37], stalk color [38], and other traits [39].

In previous studies, extensive research has been conducted on the phenotypic evaluation of cold tolerance, dividing winter rapeseed of *B. napus* into cold-tolerant and cold-sensitive varieties [40]. Therefore, in this study, we selected the cold-tolerant variety ‘16VHNTS158’ and the cold-sensitive variety ‘Tianyou 2288’ as parents to construct the genetic population, as there was a significant difference in hypocotyl length between the two (Figure 3A,C). Through SSR marker analysis, we identified a QTL associated with hypocotyl length on chromosome C04, with an additive effect of 0.025. This locus explained 2.5% of the phenotypic variation (Figure S2). Currently, there are limited reports on hypocotyl-length QTL in *B. napus*, but relevant studies have been conducted in other species. Previous studies on cold tolerance QTL in Medicago truncatula have been identified, in which two QTLs controlling cell number and three QTLs controlling cell length at low temperature were detected. One QTL for cell number and two for cell length were found to be associated with hypocotyl length under low temperature [41]. In soybean, two QTLs for hypocotyl length (qHYL1-2) were identified on linkage groups with peak LOD scores of 2.51 and 2.85 and R2 of 39.54 and 39.21%, respectively [24]. Through BSA analysis, SNPs related to hypocotyl length were detected (Figure 5A). In total, we identified 1,781,206 SNPs in ‘16VHNTS158’, 1,861,149 SNPs in ‘Tianyou 2288’, and 3,364,057 SNPs in the L-pool. Additionally, we identified 2,580,023 SNPs in the S-pool, with a similar distribution to ‘16VHNTS158’. Furthermore, we identified 17,977 Indels in ‘16VHNTS158’, 18,079 Indels in ‘Tianyou 2288’, 33,511 Indels in the L-pool, and 25,070 Indels in the S-pool. Through association analysis, we identified a total of 20 non-synonymous SNPs between the parents and 26 non-synonymous SNPs within the pooled samples. Moreover, there were six frameshift Indels between the parents and seven frameshift Indels within the pooled samples. The target trait region was localized on chromosome C04, with a total length of 1.06 Mb (Figure 5B). Within this region, we also identified 24 candidate genes, which require further investigation of their functions in subsequent studies. The hypocotyl trait is one of the components of yield in winter rapeseed (*Brassica napus* L.). The proposed QTLs are of significant importance for the breeding of cold-tolerant varieties and germplasm in winter rapeseed.

## 4. Materials and Methods

### 4.1. Experiment Population

Based on the previous screening of cold tolerance varieties in our laboratory, we selected the short hypocotyl cold-resistant variety ‘16VHNTS158’ and the long hypocotyl cold-sensitive variety ‘Tianyou 2288’ as the parents to construct F_2_ segregating populations (differences among the parents are shown in Table S5). The autumn sowing experiment (F_2_-1) was conducted on 25 August 2022, in Shangchuan Town, Lanzhou City, Gansu Province, China. The population consisted of 185 individual plants and was utilized for genetic analysis, BSA-seq, and SSR marker polymorphism detection. Each F_2_ individual was sown in 5 rows, with a row length of 200 cm, a plant spacing of 10 cm, and a row spacing of 20 cm. At the 5-leaf stage, tillering stage, stem elongation stage, initial flowering stage, and maturity stage, the length of the peduncle was measured for each individual plant using a vernier caliper.

The spring sowing experiment (F_2_-2) was conducted on 29 March 2023, in Shanchuan Town, Lanzhou City, Gansu Province, China. The experiment was carried out using a pot culture method. Clean and plump seeds were selected and germinated on a cultivation dish with two layers of filter paper as the germination bed (14 h of light at 25 °C and 10 h of darkness at 20 °C). After the seeds showed signs of germination, they were transplanted into pots filled with substrate for further cultivation. Three pots were allocated for each of the two parents, three pots for F_1_ population, and forty pots for the F_2_ population. Each pot contained 8 seedlings. During the sowing process, consistent lighting conditions, soil quantity and height, seed sowing depth, and watering volume were maintained. The plants were cultivated until the 5-leaf stage and were used for the assessment of cold resistance indicators, such as the LT50, ion leakage rate, dead cell area, and hormone determination. As for the cold acclimation treatment, the plants at a growth stage of around 5–6 leaves were placed in a natural environment for 3 days and then transferred to a growth chamber with a temperature of 4 °C for cold acclimation for an additional 3 days. Subsequently, plants were subjected to dark cold treatment either in a low-temperature circulator or a sub-zero temperature growth chamber. The treatment involved lowering the temperature by 2 °C every 30 min from 0 °C until reaching the desired temperature, followed by a continuous treatment at that temperature for 1 h.

### 4.2. Determination of LT50 and Ion Leakage Rate

The materials were incubated at 24 °C for 12 h and then treated at 4 °C, 0 °C, −2 °C, −4 °C, −6 °C, and −8 °C for 30 min, with three replicates set for each temperature. Logistic Equation (1) was utilized to fit the treatment temperature to the cell injury rate, where Y represents the ion leakage rate, t represents the treatment temperature, k represents the maximum injury rate (as the ion leakage rate removes background interference, the value of k is 100% in this experiment), and a and b are the parameters of the equation [42]. To determine the values of a and b, Equation (2) was linearized, such that $Y=\ln\left[\frac{k-Y}{Y}\right]$, which translates into a linear equation that transforms the rate of cell injury (y) with the treatment temperature (t) [43]. The values of parameters a and b and the correlation coefficient r were obtained through linear regression [44]. To determine the LT50, the second-order derivative of the logistic equation was taken and made equal to 0 to obtain the inflection point of the curve t=lna/b, where the value of t is the lethal temperature (LT50) [45].

(1) Y=k/(1+ae^(−bt)),

(2) $$\ln\left[\frac{k-Y}{Y}\right]=\ln\left(a\right)-bt,$$

The materials and treatments are the same as those given in Section 4.2. The method for the determination of ion leakage rate was based on the method of Li et al. [46], with slight modifications. The main steps are as follows: (1) Take 5-leaf stage for testing using a puncher and place in a 5 mL centrifuge tube, add 4 mL ddH_2_O, and shake gently on a horizontal shaker for 30 min. (2) Determine the conductivity of the above centrifuge tubes with a conductivity meter in the ddH_2_O, and record it as S_0_ and S_1_, respectively. (3) Put the 5 mL centrifuge tube with the leaf samples in a sterilizer and boil it at 100 °C for 10 min. Allow the tube to cool to room temperature and gently shake it on a horizontal shaker for 1 h, which is recorded as S_2_. Ion leakage rate $=\frac{S1-S0}{S2-S0}\times 100\%$.

### 4.3. Observation of Trypan Blue Staining and Counting of Dead Cell Area

A total of 10 mL glycerol, 10 mL lactic acid, 10 mL phenol, and 10 mL sterile water were mixed, and 10 mg Trypan Blue powder was added to make Trypan Blue staining solution. The leaves of the same part of each species were cut into 1 cm × 1 cm squares and placed in 10 mL centrifuge tubes, and 5 mL of dye solution (95% anhydrous ethanol: Trypan Blue = 2:1, *v*/*v*) was added and boiled for 15 min after 10 min of resting, then rinsed with sterile water, and an appropriate amount of chloral hydrate (2.5 mg/mL) was added to decolorize the leaves in a shaker overnight. When the leaves had been decolorized until they were transparent, they were placed under a stereomicroscope for photographs and observation, and the dead cell areas of ‘16VHNTS158’ and ‘Tianyou 2288’ after different low-temperature stresses were counted using Image J 2.15.0.

### 4.4. Measurement of Endogenous Hormone Content

For a description of the endogenous hormone extraction method, refer to the method of Müller et al. [47], though ours featured slight modification. When the plant grew to the 5-leaf stage, all the potted materials were placed in the 22 °C incubator for 48 h and then placed in the incubator at room temperature (22 °C), 4 °C, 0 °C, and −4 °C, respectively, with samples taken at intervals of 24 h. Over 2 g of tissue from hypocotyl was used for plant hormone quantification using three biological replicates per treatment. A total of 50 mg of powder was extracted with 1 mL of n-propanol: water: hydrochloric acid (2:1:0.002, *v*/*v*/*v*) solution containing 0.001 ng of an internal standard. After vortexing, centrifuging, and concentrating it, the concentrated sample was dissolved in 1 mL of a 50% methanol and water solution. Finally, each sample was filtered through a 0.22 µm microporous membrane for high-performance liquid chromatography (HPLC) analysis. 6-BA, ABA standard (purity ≥ 98%), and GA standard (purity ≥ 90%) were purchased from Shanghai Yuanye Biotechnology Co., Ltd. (Shanghai, China). Methanol, ethyl acetate (chromatographic purity, Gansu Elvey Scientific Instrument Co., Ltd. (Lanzhou, China)), and glacial acetic acid (chromatographic purity, Tianjin Kaixin Chemical Industry Co., Ltd. (Tianjin, China)) were also used. All the water used in the test was ultrapure; other reagents were domestic analytical pure. Determination was carried out using a Waters Arc-type high-performance liquid chromatograph (Shanghai, China). Chromatographic column: Agilent ZORBAX Eclipse Plus C18 column (4.6 mm × 250 mm, 5 μm); mobile phase: methanol water (45:55, *v*/*v*); column temperature: 30 °C; detection wavelength: 254 nm; flow rate: 1 m L·min^−1^; injection volume: 10 μL.

### 4.5. Mixed Pool Construction and Sequencing Data Processing

Construction of parent pools: 10 individuals of short hypocotyl parent ‘16VHNTS158’ and 10 individuals of long hypocotyl parent ‘Tianyou 2288’ were mixed to form the parent pool. Construction of progeny pools was as follows: two pools, named the S-pool and L-pool, were created with 30 individuals each from the F_2_-1 population displaying short and long hypocotyl phenotypes, respectively.

Equal amounts of DNA from the parent and progeny pools were mixed to create two parent pools and two progeny pools, which were then sent to Beijing Baimaike Biotechnology Co., Ltd. (Beijing, China) for sequencing analysis. The DNA was sonicated to generate fragments with an insert length of 350 bp. After passing the library quality inspection, the libraries were subjected to sequencing using the Illumina HiSeq platform. The sequencing depth for the parent pool was 10×, while the progeny sequencing depth was 30×.

The Euclidean distance (ED) was used to calculate the Euclidean distance between the two groups of samples, and the SNP index of the extreme mixed pool of the two offspring was filtered out to be less than 0.3, and only the SNP loci that were biparentally pure and different, and the pools of the two offspring that were non-missing were retained. Delta SNP index was obtained by subtracting the SNP index of the two offspring pools, and the higher the value of Delta SNP index, the stronger the association between the marker SNP and the trait, and the gene in which the locus is located is likely to be related to the target trait. For the ED values of SNP or Indel markers on the same chromosome, regression fitting of LOESS was performed to obtain the threshold of association, and the region above the 99% quantile threshold was selected as the region of association relaxed with the trait. The original raw reads were generated after excluding low-quality reads and adapter sequences using fastp [48]. The unique reads were aligned to the *B. napus* reference genome new version (http://plants.ensembl.org/Brassica_napus/Info/Index, accessed on 3 June 2023) using HISAT2 v 2.1.0 with default parameters [49].

### 4.6. DNA Extraction and PCR Amplication

Genomic DNA was extracted from leaves using a Plant Genomic DNA Extraction Kit (product number DP305) from TIANGEN Biotech (Beijing, China) according to the manufacturer’s instructions. Meanwhile, 64 polymorphic SSR markers in the rapeseed genome database (http://brassicadb.cn, accessed on 15 May 2023) were selected, and the primers were synthesized and used as templates for PCR amplification of leaf DNA. The scanning gel films in the F_2_-1 population were analyzed statistically. The markers that matched the bands of the maternal parent ‘16VHNTS158’ were designated as “A”, while those matching the bands of the paternal parent ‘Tianyou 2288’ were designated as “B”. Heterozygotes were labeled as “H”, and missing or unclear bands were denoted as “-”.

PCR amplifications were performed in 10 μL reactions, each containing 2 μL template DNA (50 ng/μL), 0.5 μL forward primers (5 μmol/L), 0.5 μL reverse primers (5 μmol/L), 2 μL ddH_2_O, and 5 μL 2× Taq PCR Master mix (Tiangen Biotech Co., Ltd., Beijing, China). The PCR program was as follows: 4 min at 95 °C, 40 s denaturing at 94 °C, 40 s annealing at 60 °C, and 45 s elongation at 72 °C, followed by a 1 °C reduction in the annealing temperature per cycle for 10 cycles. Then, annealing temperature was reduced in each cycle by 1 °C for 8 cycles from 55 °C; the annealing temperature was maintained at 50 °C for the remaining 30 cycles, followed by a final step at 72 °C for 5 min. The amplified PCR products were separated via vertical electrophoresis in 8% polyacrylamide gel in 1 × TBE buffer at a constant 180 V for 1.5 h, visualized with silver staining and captured using a digital camera [50].

### 4.7. Statistical and Functional Candidate Genes Analyses

#### 4.7.1. Statistical Analyses

All figures are based on Origin2021. Statistical analysis and genetic models are based on IBM SPSS Statistics 23 and Microsoft® Excel® 2016MSO (16.0.16827.20166). Cell death area is based on Image J 2.15.0. Genetic population analysis was performed using R software package (version 2.0.1). Construction of genetic linkage mapping and QTL localization was carried out using QTL IciMapping 4.0.

#### 4.7.2. Functional Annotation of Candidate Genes

The coding genes within the candidate regions were subjected to comprehensive annotation using the BLAST software 2.14.0 [51] against multiple databases, including NR [52], Swiss Prot [53], GO [54], and KEGG [55]. Candidate genes were rapidly screened through detailed annotation.

## 5. Conclusions

In this study, BSA-seq localized the primary QTL locus to chromosome C04 and associated it with 41 regions with a total length of 1.06 Mb, which was verified by SSR markers. The LT50 and ion leakage rate of short hypocotyl plants were lower than those of long hypocotyl plants, and the contents of the endogenous hormones IAA and GA were higher than those of long hypocotyl plants. The study of this trait will provide a more genetic and physiological basis for the breeding of cold resistance in *B. napus*.

## Figures and Tables

**Table 1:** Relative conductivity of leaves and logistic fitting equation.

Variety	Relative Electric Conductivity (%)							Regression Equation	R2	LT50 (°C)
	4 °C	0 °C	−2 °C	−4 °C	−6 °C	−8 °C	−10 °C			
16VHNTS158	4.1 ± 0.1	17.7 ± 0.1	20.7 ± 0.1	32.0 ± 0.1	38.5 ± 0.4	48.3 ± 0.4	65.4 ± 0.2	Y = 1.8786 − 0.250X	0.97	−7.5
Tianyou 2288	6.8 ± 0.01	18.1 ± 0.1	25.8 ± 0.1	38.2 ± 0.3	55.3 ± 0.2	62.3 ± 0.1	83.2 ± 0.5	Y = 1.5499 − 0.288X	0.99	−5.4

**Table 2:** Phenotypic statistical analysis of hypocotyl length in parental and F_2_-1 populations at 5-leaf stage.

Population	Average Length (cm)	Max	Min	Range	CV (%)	Kurtosis	Skewness
F_2_	0.51	1.5	0	1.5	46	2.42	0.64
16VHNTS158	0.41	0.7	0	0.7	37	3.02	−1.1
Tianyou 2288	0.77	1.2	0.4	1.2	30	−0.87	0.3

**Table 3:** AIC value and optimal genetic model for hypocotyl length in the F_2_-1 population.

Model	AIC	$U_{1}^{2}$	$U_{2}^{2}$	$U_{3}^{2}$	nW^2^	Dn
1MG-AD	263.2167	0.128 (0.96)	0.0784 (0.85)	0.0704 (0.35)	0.1123 (0.16)	0.0646 (0.03)
1MG-A	269.9081	0.0022 (0.93)	0.0349 (0.86)	0.8623 (0.69)	0.2786 (0.32)	0.1114 (0.15)
1MG-EAD	272.2328	0.0069 (0.70)	0.0322 (0.72)	0.1575 (0.97)	0.1744 (0.14)	0.0876 (0.03)
1MG-NCD	290.9928	0.1468 (0.87)	0.1308 (0.85)	0.0014 (0.16)	0.302 (0.08)	0.1105 (0.02)
2MG-ADI	294.5386	0.0281 (0.97)	0.0347 (0.95)	1.9439 (0.94)	0.4003 (0.74)	0.1198 (0.74)
2MG-AD	247.2805	0.0016 (0.87)	0.0032 (0.97)	0.0052 (0.42)	0.0736 (0.60)	0.0528 (0.47)
2MG-A	247.3956	0.0287 (0.37)	0.0014 (0.55)	0.6458 (0.26)	0.0998 (0.18)	0.0653 (0.03)
2MG-EA	259.385	0.808 (0.55)	0.3494 (0.57)	1.2482 (0.97)	0.2654 (0.11)	0.114 (0.01)
2MG-CD	294.5331	0.3587 (0.55)	0.325 (0.57)	0.0015 (0.97)	0.3373 (0.11)	0.1223 (0.01)
MG-EAD	292.5332	0.3587 (0.55)	0.325 (0.57)	0.0015 (0.97)	0.3372 (0.11)	0.1223 (0.01)

Note: AIC: AIC =−2L(Y|Θ)+2k); L (Y|Θ): logarithm likelihood function, parameter in the logarithm likelihood function; k: the number of independent parameters in the model; $U_{1}^{2}$, $U_{2}^{2}$, $U_{3}^{2}$: uniform; nW^2^: Smirnov Dn: Kolmogorov statistics. P ($U_{1}^{2}$), P ($U_{2}^{2}$), P ($U_{3}^{2}$), P (nW^2^), P (Dn): *p*-values of the above corresponding statistics.

**Table 4:** Estimation of genetic parameters for different genetic models.

	Model	1MG-AD	1MG-A	1MG-EAD	2MG-AD	2MG-A	2MG-EA
First-order genetic parameter	M	0.78	0.98	0.83	1.31	1.00	1.04
	d_a_	0.26	0.40	0.30	0.40	0.22	0.29
	d_b_	-	-	-	0.46	0.44	-
	h_a_	0.48	-	-	−0.37	-	-
	h_b_	-	-		−0.30	-	-
	I	-	-	-	-	-	-
	j_ab_	-	-	-	-	-	-
	j_ba_	-	-	-	-	-	-
	L	-	-	-	-	-	-
Second-order genetic parameter	Major-Gene Var	0.195					
	Heritability (%)	59.54					

Note: M, d, h: total average, additive effect, and dominant effect for major gene; i, j_ab_, j_ba_, l: additive × additive, additive × dominance, dominance × additive, and dominance × dominance interaction effects between two major genes. Major-Gene Var, Polygenes Var: genetic variances for major genes and polygenes (second-order genetic parameter), respectively. Heritability (%): the proportion of genetic variance in total phenotypic variance.

**Table 5:** Sample comparison results.

Name	Mapping Reads	Mapping Ratio (%)	Ave Depth	Properly Paired Ratio (%)	Q20%	Q30%	GC (%)
16VHNTS158	141,541,608	99.15	23	88.85	97.65	93.42	36.51
Tianyou 2288	146,156,508	98.97	23	88.39	97.17	92.38	36.69
S-pool	199,081,960	99.47	32	92	97.38	93.44	37.35
L-pool	203,088,138	99.22	33	90.76	97.99	92.21	37.34

Note: S-pool represents a short hypocotyl pool; L-pool represents a long hypocotyl pool.

## Data Availability

The data presented in this study are available in this article and the Supplementary Materials.

## Supplementary Materials

The following supporting information can be downloaded at: https://www.mdpi.com/article/10.3390/ijms242015409/s1.
