# Supplementary material for: Physiological Analysis and Genetic Mapping of Short Hypocotyl Trait in Brassica napus L
Source: Int J Mol Sci. 2023 Oct 21;24(20):15409. doi: 10.3390/ijms242015409 (PMC10607371; doi:10.3390/ijms242015409)
Supplement: Supplementary file 1 [file ijms-24-15409-s001.zip › ijms-2638654-supplementary.pdf]

Supplementary Materials

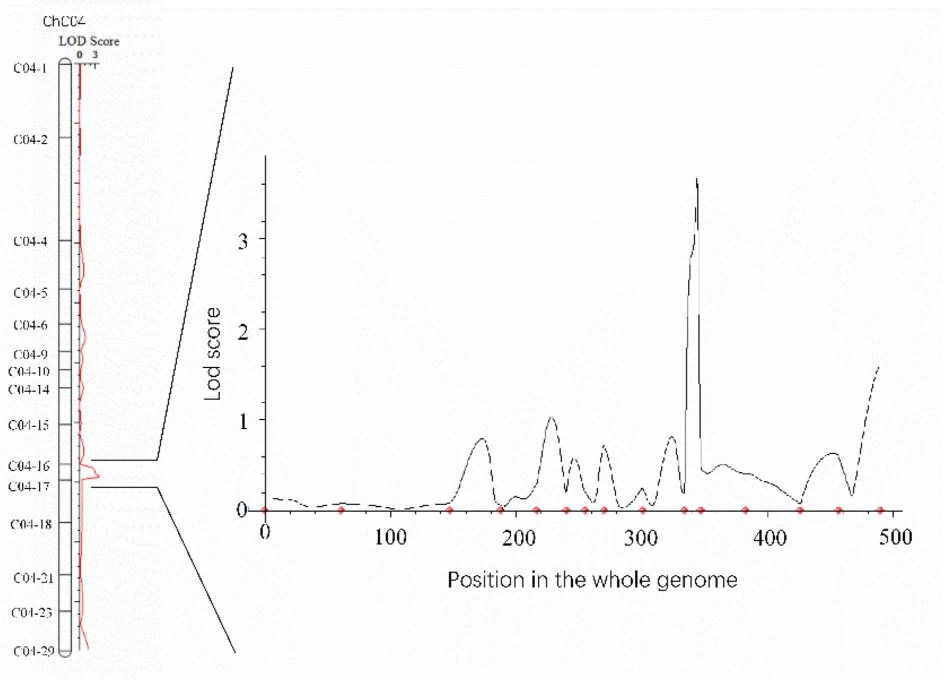

Figure S1. QTLs detected in hypocotyl length of the F2-1population.

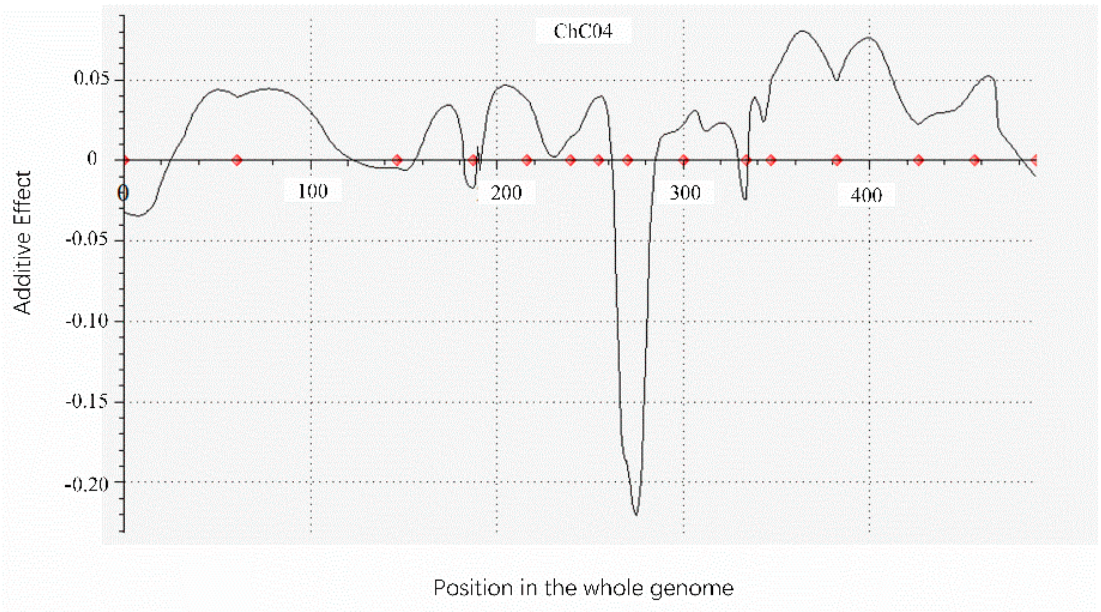

Figure S2. Additive effect values of the QTL loci on hypocotyl length.

Table S1. Correlation analysis between hypocotyl length and cold resistance of winter rapeseed (Brassica napus).

	Hypocotyl Length	LT50	Area Dead Cell	Ion Leakage Rate
Hypocotyl length	1			
LT50	0.814 **	1		
Area dead cell	0.915 **	0.764 **	1	
Ion leakage rate	-0.437 **	-0.179	-0.365 **	1

Note: Asterisks indicate significant differences: ** $p < 0.01$

Table S2. Statistical table for information on the associated region.

Chromosome_ID	Start	End	Size (Mb)
LK031793	1473992	1474511	0.00
LK031793	1474635	1474668	0.00
LK031793	1474790	1478450	0.00
LK031793	1479622	1479622	0.00
LK031793	1480328	1480328	0.00
LK031793	1480473	1480989	0.00
LK031793	1484623	1489764	0.01
LK031793	1491309	1492057	0.00
LK031793	1499293	1545516	0.05
LK031793	1679257	1679257	0.00
LK031793	1722948	1722948	0.00
LK031793	1792465	1792465	0.00
LK031793	1837729	2122910	0.29
LK031793	2883037	3008003	0.12
LK031793	294581	297152	0.00
LK031793	299019	299149	0.00
LK031793	300646	302199	0.00
LK031793	30379	286304	0.26
LK031793	3055394	3262536	0.21
LK031793	631834	632883	0.00
LK031793	633073	633073	0.00
LK031793	645986	646857	0.00
LK031793	648257	650353	0.00
LK031793	681437	681437	0.00
LK031793	684145	685067	0.00
LK031793	685427	723382	0.04
LK031793	724241	725612	0.00
LK031793	796633	796788	0.00
LK031793	798225	798839	0.00
LK031793	799011	799564	0.00
LK031793	801287	803162	0.00
LK031793	805779	811031	0.01
LK031793	811195	856949	0.05
LK031793	857772	857925	0.00
LK031793	858272	859022	0.00
LK031793	859136	859259	0.00
LK031793	859334	884020	0.02
LK031793	884421	886900	0.00
LK031793	891312	891433	0.00
LK031793	897668	897668	0.00
LK031793	898677	898677	0.00
Total	-	-	1.06

Table S3. Distribution of SSR markers on different linkage groups.

Linkage Group	LOD Value	Marker Number	Length (cM)	Average Distance(cM)
C04	2.5	15	489.71	32.65

Table S4. The functional annotations of the candidate genes.

Gene ID	Start	End	Function
BnaC04g16150D	292265	295128	Coiled-coil SMC6 And NSE5 INteracting (CANIN) domain
BnaC04g15940D	49664	50472	Cyclin-dependent protein kinase inhibitor SMR11/SMR16
BnaC04g15950D	61687	62455	Cyclin-dependent protein kinase inhibitor SMR11/SMR16
BnaC04g15960D	62746	67402	2, C-terminal disease resistance protein
BnaC04g15970D	77623	79448	Sugar phosphate transporter domain
BnaC04g15980D	80236	82877	Protein of unknown function
BnaC04g15990D	96068	100565	Cytokinin riboside 5'-monophosphate phosphoribohydrolase LOG
BnaC04g16000D	146721	149986	Ion transport domain
BnaC04g16010D	165034	168123	Protein kinase domain
BnaC04g16020D	169496	171958	Protein of unknown function
BnaC04g16030D	172230	173368	Mediator complex, subunit Med20
BnaC04g16040D	176402	178003	Alpha carbonic anhydrase
BnaC04g16050D	183579	184919	Protein of unknown function
BnaC04g16060D	195147	195458	Protein of unknown function
BnaC04g16070D	202320	204861	Replication factor A, C-terminal
BnaC04g16080D	212270	213501	Replication factor A, C-terminal
BnaC04g16090D	213566	214483	Replication factor A protein 1
BnaC04g16100D	235505	236658	Protein of unknown function
BnaC04g16110D	242700	243847	Protein of unknown function
BnaC04g16120D	259788	261391	Myc-type, basic helix-loop-helix (bHLH) domain
BnaC04g16130D	264391	267064	Protein SOSEKI

Table S5 Comparison of cold tolerance differences in parents.

Varieties	Material source	Hypocotyl length(cm)	vernalization rate(%)	Over-wintering rate（%）
16VHNTS158	Gansu Agricultural University	0.54	80.17	40.56
Tianyou 2288	Tianshui agricultural institute	1.53	94.21	10.3
